# Supplementary material for: Searching for SARS-CoV-2 in Cancer Tissues: Results of an Extensive Methodologic Approach based on ACE2 and Furin Expression
Source: Cancers (Basel). 2022 May 24;14(11):2582. doi: 10.3390/cancers14112582 (PMC9179515; doi:10.3390/cancers14112582)
Supplement: Supplementary file 1 [file cancers-14-02582-s001.zip › cancers-1697047-supplementary.pdf]

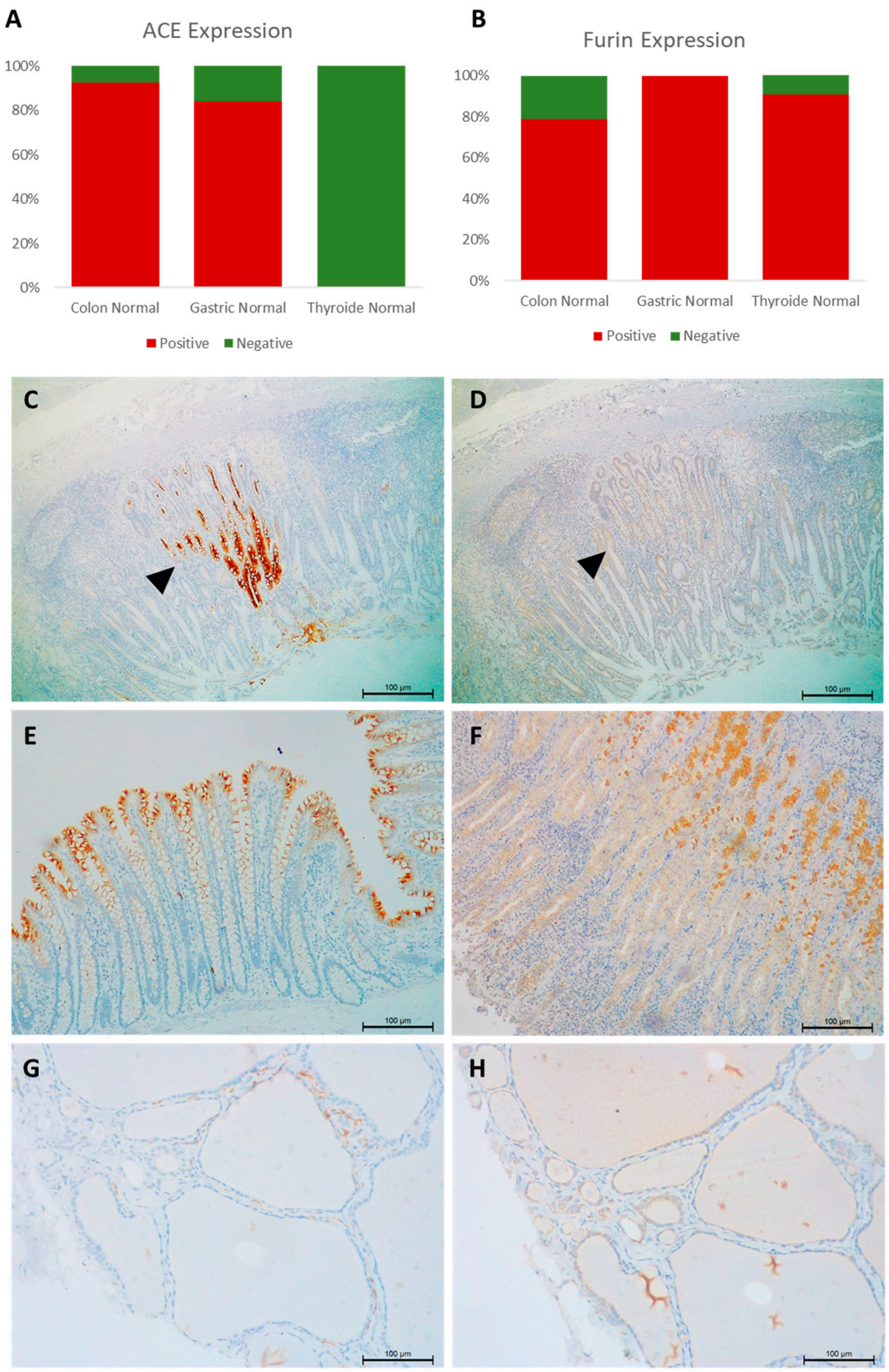

Supplementary Figure S1. ACE2 and Furin expression in areas of normal tissues adjacent to carcinoma of thyroid, stomach and colon. The graphics A and B represent the percentage of positive and negative cases for the expression of ACE2 and Furin in the different carcinomas. ACE2 was highly expressed in areas of intestinal metaplasia (arrowhead) in the stomach (C) and in normal colon mucosa (E), but was not observed in normal thyroid follicular cells (G). Furin expression was present in normal adjacent areas of the stomach (D), colon (F) and thyroid (H).

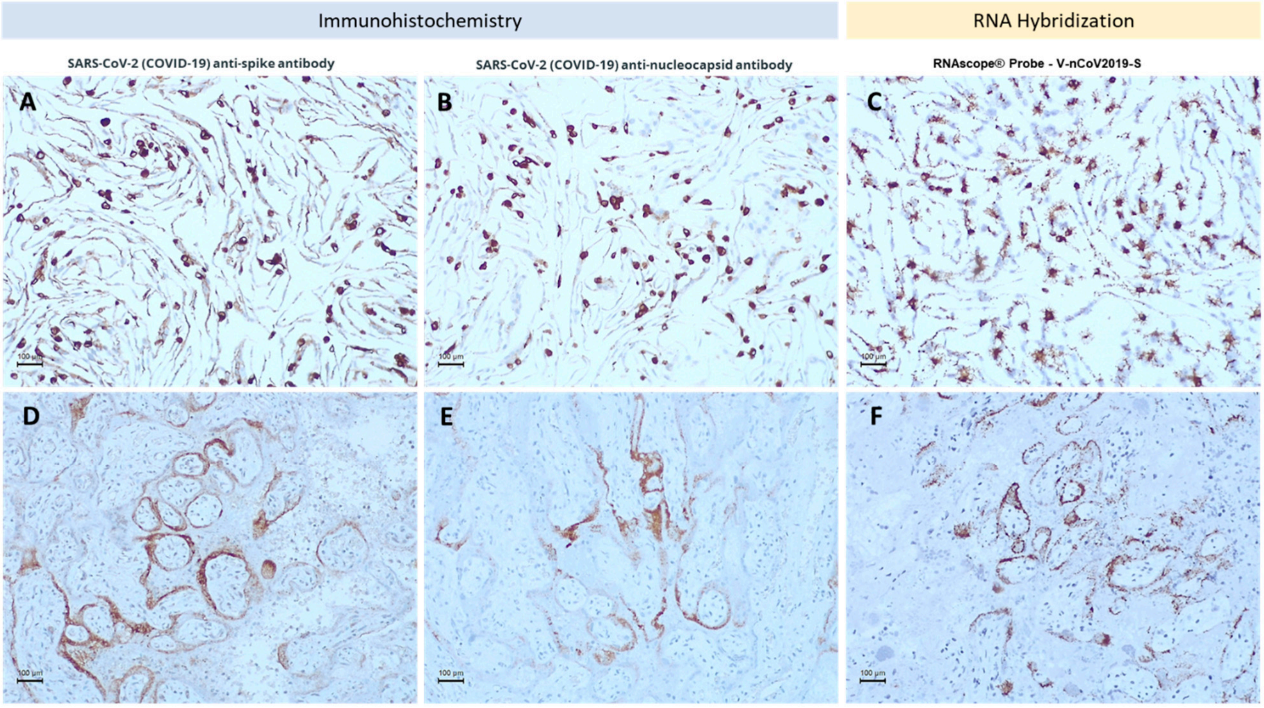

Suplementary Figure S2.VERO Cells Infected with SARS-CoV-2 stained with SARS-CoV-2 (COVID-19) spike antibody [1A9] (GeneTex) (A), SARS-CoV-2 (COVID-19) nucleocapsid antibody [6H3] (GeneTex) (B) and RNAscope® Probe - V-nCoV2019-S (C). Human placenta sample from a SARS-CoV-2 infected patient stained with SARS-CoV-2 (COVID-19) spike antibody [1A9] (GeneTex) (D), SARS-CoV-2 (COVID-19) nucleocapsid antibody [6H3] (GeneTex) (E) and RNAscope® Probe - V-nCoV2019-S (F).

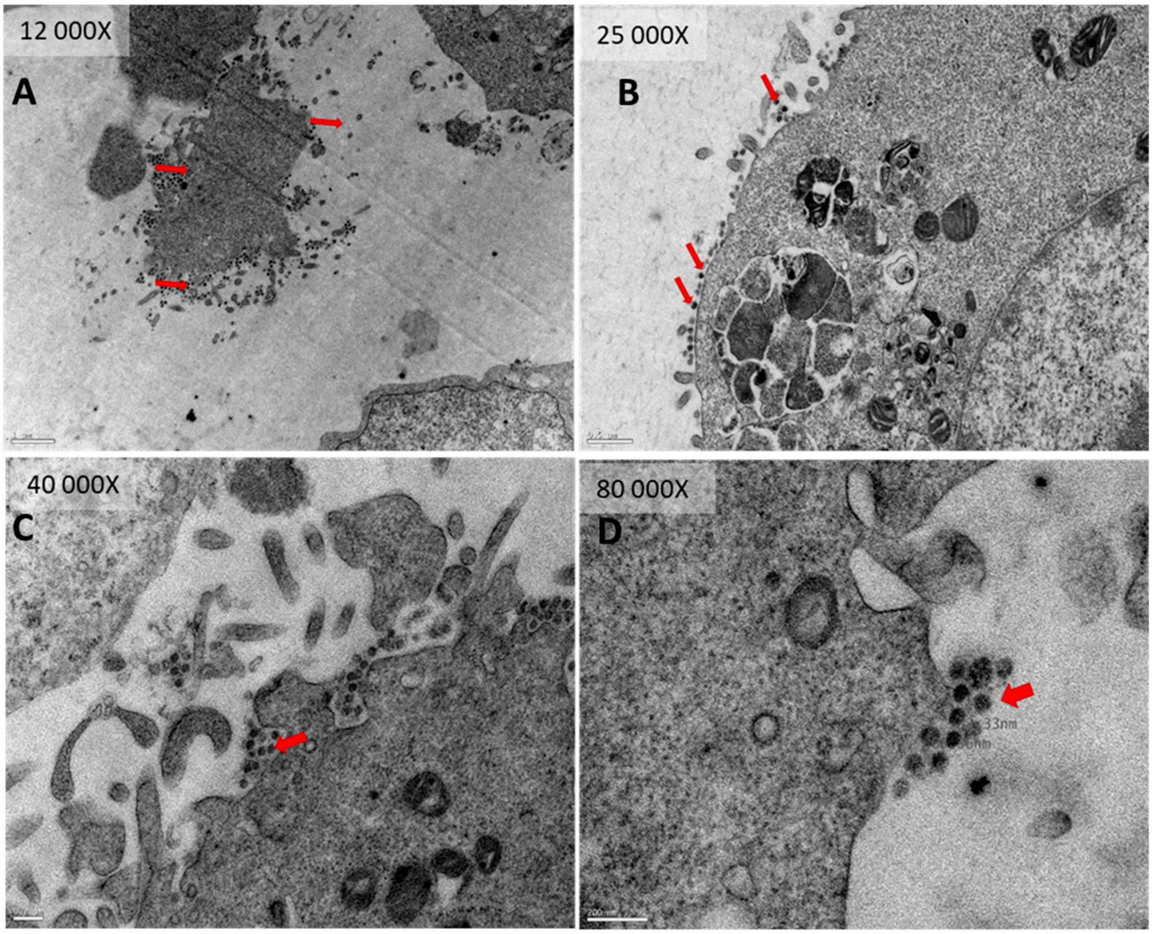

Supplementary Figure S3. VERO Cells infected with SARS-CoV-2. Transmission electron microscopy shows SARS-CoV-2 viral particles (red arrow) at the cell membrane and in the cytoplasm in aggregates and in vesicles, observed with 12 000x magnification (A), with 25 000x magnification (B), with 40 000x magnification (C) and, with 80 000x magnification (D).

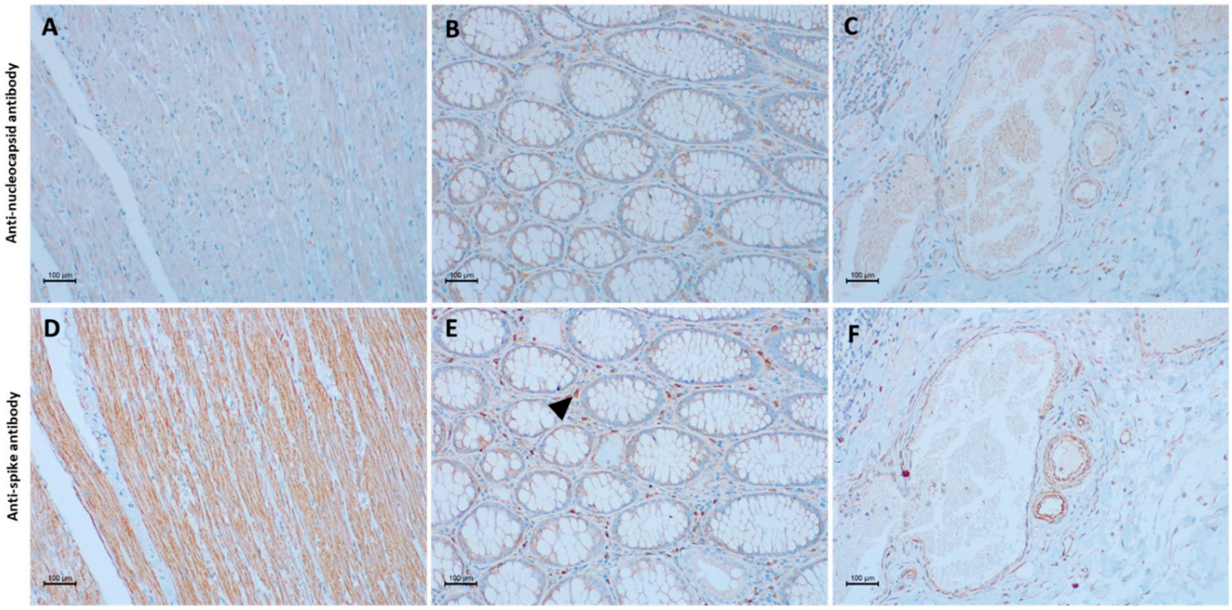

Supplementary Figure S4. Immunostaining for spike and nucleocapsid SARS-CoV-2 proteins in normal tissues adjacent to tumor areas. Anti-spike and anti-nucleocapsid primary antibodies showed unspecific staining in normal tissue components such as smooth muscle (A and D), lamina propria inflammatory cells (B and E) and blood vessels (C and F)

Supplementary Table S1. Association between the staining results obtained with two different antibodies anti-SARS-COV-2.

		**Anti-spike protein antibody**		
		**Positive (%)**	**Negative (%)**	**p value**
**Anti-nucleoprotein antibody**	**Positive**	4 (80.0%)	2 (15.4%)	0.022
	**Negative**	1 (20.0%)	11 (84.6%)	
